# A case of a lesion containing an intracoronary thrombus detected as hyperintense plaque on T1-weighted cardiovascular magnetic resonance in a patient with silent myocardial ischemia

**DOI:** 10.1186/1532-429X-15-50

**Published:** 2013-06-13

**Authors:** Kenji Matsumoto, Shoichi Ehara, Takao Hasegawa, Kenichiro Otsuka, Takanori Yamazaki, Tomokazu Iguchi, Kenei Shimada, Minoru Yoshiyama

**Affiliations:** 1 Department of Internal Medicine and Cardiology, Osaka City University Graduate School of Medicine, Osaka, Japan

**Keywords:** Coronary artery disease, Cardiovascular magnetic resonance, Thrombus, Optical coherence tomography

## Abstract

Many investigators have speculated that hyperintense plaques (HIPs) of the carotid artery on noncontrast T1-weighted imaging (T1WI) in cardiovascular magnetic resonance indicate the presence of mural or intraplaque hemorrhage containing methemoglobin. However, coronary plaque imaging with T1WI is challenging, and the clinical significance of coronary HIPs on T1WI remains unknown. Incidentally, it is very rare to find an intracoronary thrombus at the culprit lesion site in patients in stable condition. This article reports the case of a lesion containing an intracoronary thrombus, detected as HIP on T1WI associated with the filter no-reflow phenomenon in a patient with silent myocardial ischemia.

## Background

Many investigators have speculated that hyperintense plaques (HIPs) of the carotid artery on noncontrast T1-weighted imaging (T1WI) in cardiovascular magnetic resonance (CMR) indicate the presence of mural or intraplaque hemorrhage containing methemoglobin [1,2]. Coronary plaque imaging with T1WI is challenging, and the clinical significance of coronary HIPs on T1WI remains unknown. Jansen et al. demonstrated that within 72 hours after the initial onset of symptoms, 10 of 18 patients with acute myocardial infarction (AMI) were found to have intracoronary thrombi as detected by invasive coronary angiography, and that HIPs on T1WI correctly identified the intracoronary thrombi [3]. Recently, we have shown that intraluminal HIPs on T1WI are related to the presence of intracoronary thrombi as detected by optical coherence tomography (OCT) imaging [4].

Previous studies by using intravascular ultrasonography [5] or OCT [6] showed that multiple unstable plaques, associated with plaque rupture, thin-cap fibroatheroma (TCFA), and intracoronary thrombi, were more common in patients with AMI than in those with stable angina pectoris (SAP). Recently, Kubo et al. demonstrated that intracoronary thrombi were not found in the culprit and nonculprit lesions of SAP patients, though they were observed in 100% and 8% of the culprit and nonculprit lesions, respectively, of AMI patients [6]. Thus, it is very rare to find an intracoronary thrombus at the culprit lesion site in patients in stable condition.

This article reports the case of a lesion containing an intracoronary thrombus, detected as HIP on T1WI associated with the filter no-reflow phenomenon in a patient with silent myocardial ischemia.

## Case presentation

**Figure 1:**
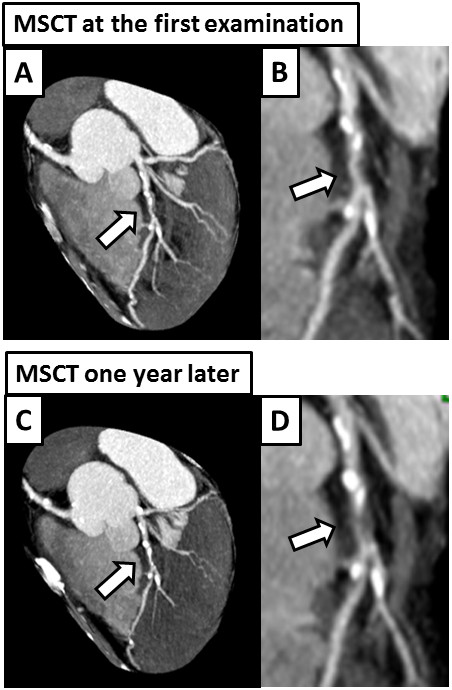
**MSCT coronary angiography at the first examination (A and B) and one year later (C and D). A**, At the first examination, MSCT coronary angiography showed moderate stenosis (indicated by arrow) in the mid LAD (partial-width maximum intensity projection). The area of coronary stenosis is shown in higher magnification in panel **B** (maximum intensity projection). **C** and **D**, One year later, follow-up MSCT coronary angiography showed a slight progression of coronary stenosis.

**Figure 2:**
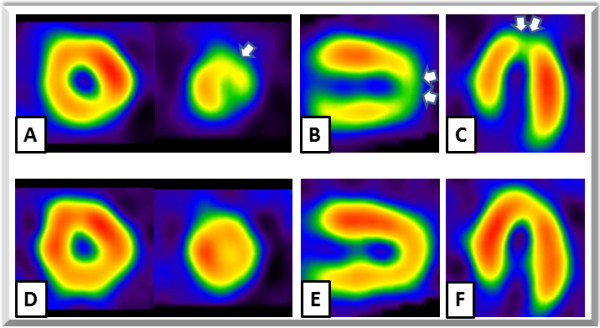
**The exercise stress thallium-201 single-photon emission computed tomography.** The exercise stress images (**A-C**) showed moderate defect in apico-anterior wall. The images at rest (**D-F**) showed redistribution in the same area. They revealed apico-anterior myocardial ischemia.

**Figure 3:**
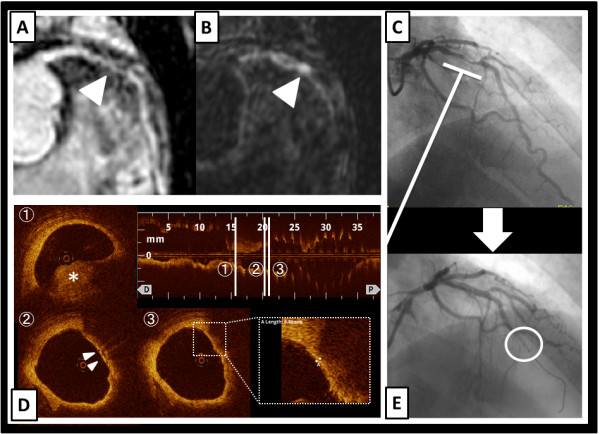
**The culprit lesion in the mid LAD. A**, Whole-heart coronary MR angiographic image showed moderate coronary stenosis (indicated by arrowhead) in the mid LAD. **B**, Coronary T1WI image showed HIP (indicated by arrowhead) in the area corresponding to the moderate stenotic lesion. **C**, Coronary angiographic image showed moderate coronary stenosis in the mid LAD. **D**, OCT images showed intracoronary thrombus (1), macrophages (2), and thin-cap fibroatheroma (3) in the culprit lesion. **E**, Coronary angiographic image showed the filter no-reflow phenomenon after stent implantation.

**Figure 4:**
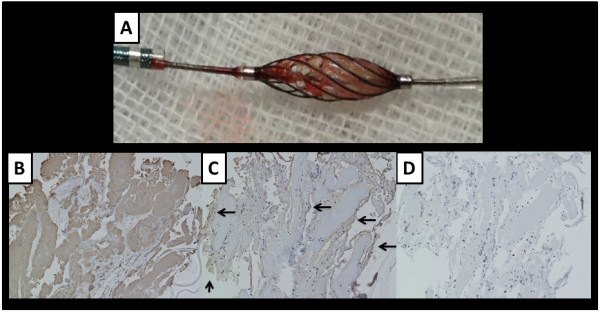
**Photograph of the filter device and Micrographs of captured debris after the procedure. A**, The filter device contained a small amount of debris. **B**, Immunostaining for platelets with anti-CD61 antibody. The specimen, stained withanti-CD61 antibody, was positive. **C**, An adjacent specimen, stained with anti-fibrinogen antibody, showed positivity for fibrinogen (indicated by arrows) to surround platelets. **D**, Immunostaining for macrophages with anti-CD68 antibody. The specimen contained CD68-positive macrophages.

A 64-year-old man with a history of dyslipidemia and current smoking had undergone multislice computed tomography (MSCT) coronary angiography to exclude coronary artery disease, although he had been fully active and denied all symptoms. At that time, MSCT coronary angiography showed moderate stenosis in the mid left anterior descending artery (LAD) (Figure 1-A, B). One year later, follow-up MSCT coronary angiography was performed, and it showed a slight progression of the coronary stenosis (Figure 1-C, D). The results of his electrocardiography, echocardiography, and cardiac enzyme testing showed no abnormalities, but exercise stress thallium-201 single-photon emission computed tomography revealed apico-anterior myocardial ischemia (Figure 2). Whole-heart coronary MR angiography, using a 1.5 T MR system (Achieva, Philips Medical Systems, Best, the Netherlands), showed moderate coronary stenosis in the mid LAD (Figure 3-A), which is the same finding as that reported by MSCT angiography. Then, the coronary plaque image was obtained when a patient was breathing freely by using a three-dimensional T1WI inversion-recovery gradient-echo sequence with black-blood condition using a Look Locker sequence, fat-suppressed and radial k-space sampling in Y-Z plane (repetition time, 4.4 ms; echo time, 2.0 ms; flip angle, 20°; SENSE factor, 2.5; number of excitations, 2; navigator gating window of ±1.5 mm with diaphragm drift correction; field of view, 300 × 240 × 120 mm (rectangular field of view, 80%); acquisition matrix, 224 × 224; reconstruction matrix, 512 × 512 × 140, resulting in an acquired spatial resolution of 1.34 × 1.34 × 1.7 mm reconstructed to 0.6 × 0.6 × 0.85 mm). The area corresponding to the stenotic lesion showed hyperintensity on noncontrast T1WI (Figure 3-B). On the next day, the patient underwent coronary angiography that showed 75% stenosis in the mid LAD (Figure 3-C) in the area corresponding to the HIP identified by T1WI. First, preinterventional OCT (ImageWire; LightLab Imaging, Inc, Westford, Massachusetts) imaging was performed on the culprit lesion in the LAD. The OCT images revealed an intracoronary thrombus, macrophages, and TCFA in the culprit lesion (Figure 3-D). Therefore, a filter-based distal protection device (Filtrap, Nipro, Japan) was deployed distal to the LAD lesion to reduce the risk of distal embolization. After stent implantation, ST-segment elevation persisted, and antegrade flow markedly diminished (Thrombolysis in Myocardial Infarction [TIMI] grade 1) (Figure 3-E). Removal of the distal protection device resulted in immediate normalization of flow with resolution of the ST-segment elevation. On inspection, the filter device contained a small amount of debris (Figure 4-A), which contained thrombus (Figure 4-B, C) and macrophages (Figure 4-D). The patient was safely discharged a few days later without any symptoms or abnormal findings on electrocardiography, echocardiography, and cardiac enzyme testing.

## Conclusions

It is unclear what caused the HIPs of the coronary artery on T1WI. There is a possibility that HIP formation detected on T1WI results from methemoglobin production, such as during intracoronary thrombus formation, intraplaque hemorrhage, or blood stasis. However, there are few reports of a lesion containing an intracoronary thrombus, detected as HIP on T1WI associated with the filter no-reflow phenomenon in a patient with silent myocardial ischemia. The present case shows that coronary HIPs on T1WI may help noninvasive identification of vulnerable plaques within the coronary arteries of the heart, regardless of the presence or absence of angina symptoms.

## Consent

Written informed consent was obtained from the patient for the publication of this case report and accompanying images. A copy of the written consent is available for review by the Editor-in-Chief of this journal.

## Abbreviations

HIP: Hyperintense plaque; T1WI: T1-weighted imaging; CMR: Cardiovascular magnetic resonance; AMI: Acute myocardial infarction; OCT: Optical coherence tomography; TCFA: Thin-cap fibroatheroma; SAP: Stable angina pectoris; MSCT: Multislice computed tomography; LAD: Left anterior descending artery.

## Competing interests

The authors declare that they have no competing interests.

## Authors’ contributions

KM was the primary author of the text. SE conceived the report, acted as the chief editor, and provided the images. TY was the primary physician during the patient’s inpatient stay. TH, KO, TI, KS, and MY were involved in the patient’s care as well as editing and overseeing of the text. All authors have read and approved the final manuscript.
